# An investigation of contextual factors in the application of multisensory illusions for analgesia in hand osteoarthritis

**DOI:** 10.1093/rap/rky019

**Published:** 2018-07-21

**Authors:** Kristy Themelis, Roger Newport

**Affiliations:** 1 Brighton and Sussex Medical School, University of Sussex, Brighton; 2 School of Psychology, University of Nottingham, Nottingham; 3 School of Sport, Exercise and Health Sciences, Loughborough University, Loughborough, UK

**Keywords:** analgesia, body ownership, body representation disturbance, chronic pain, contextual control, MIRAGE, multisensory illusions, osteoarthritis

## Abstract

**Objective:**

Emerging evidence suggests that multisensory illusions can modulate pain and can lead to changes in body perception. The aim of this study was to investigate whether contextual factors could explain the analgesic effects of multisensory body illusions on pain and body perception in people with hand OA (HOA).

**Methods:**

In a crossover study, 28 individuals with painful HOA viewed their most affected hand in and outside of a real-time mediated reality system, with illusory stretching of the hand and changes in sensory input. The outcome measures were pain ratings, pressure pain thresholds, hand function and the subjective experience of the illusion.

**Results:**

Stretching the hand both inside and outside the virtual environment led to a reduction in subjective pain ratings (all *P *< 0.05). Virtual stretching led to changes in body perception (*P* < 0.05) with no changes in pressure pain threshold (all *P* > 0.05). Higher pain at baseline predicted susceptibility to the stretch illusion and mean susceptibility ratings were greatest after the stretch illusion.

**Conclusion:**

The current study highlights the importance of the context in which pain occurs and in which potential treatments may be applied. In this case, virtual and physical stretching modulated pain, but not viewing the hand alone. The research opens important implications for future research, including the use of contextual control conditions and the development of visual feedback interventions for a range of similarly visible chronic conditions for which pain, body image disturbances and body dissatisfaction may be apparent.

**Key messages**

- Virtual and physical stretching of the hand modulates pain, but not viewing the hand alone.
- A virtual view of the painful hand leads to changes in body perception in HOA.
- Analgesic effects are not specific to the multisensory illusion and the multisensory context is important.

## Introduction

The association between radiographic signs of OA and reported pain and disability is low [1, 2], suggesting that additional underlying mechanisms are responsible. Pain in OA is associated with changes in both peripheral and central processes [1]; pressure pain thresholds (PPTs) are reduced in OA compared with controls, irrespective of whether affected, distal or remote sites are tested [3]. However, the vast majority of treatments target the periphery and there is a need for treatments that address central pain mechanisms.

Research suggests a disruption of body representation in patients with OA [4, 5] as well as in many other chronic pain conditions [6, 7]. Clinically, this may present itself in the form of body perception disturbances, in which the size or shape of the affected body part can be misperceived [8–10]. Furthermore, individuals with OA also present with high levels of body dissatisfaction, which has a major functional and psychological impact on everyday life [11].

Body perception disturbances have recently been described in OA, with changes in the perceived size of the painful body part [4]. Interventions that target body representation have demonstrated beneficial effects for several chronic pain conditions [12–14], including OA [5]. Preston and Newport [5] demonstrated that illusory stretching or shrinking of the hand, using mediated reality, can modulate pain and perceived hand size in people with hand OA (HOA). However, despite the increasing popularity of virtual multisensory interventions [13], other factors that may contribute to the observed analgesic effects are frequently overlooked. Indeed, in experimental settings, vision of the body is found to reduce pain [15–17], and several other non-specific factors, including patient expectations, clinician factors and the experimental or health care setting, are known to modulate pain perception [8, 18, 19]. Thus the current investigation directly compared the effects of multisensory illusions on pain and body perception with other combinations of multisensory information as well as contextual factors that may contribute to the observed analgesic effects.

The proposed research intended to assess whether such applications might also affect pain sensitivity using static quantitative sensory testing of PPTs before and after multisensory illusions designed to modulate pain in HOA. Additionally, the study investigated the effects on hand function, based on previous anecdotal observations indicating improved hand mobility and range of motion after illusory stretching of the affected hand [5].

## Materials and methods

### Participants

Thirty adults with a diagnosis of HOA were recruited through the university database and via printed advertisements. Power analysis for repeated measures analysis of variance (ANOVA) was conducted using G*Power [20] to determine the required sample size to test the main effect of condition on numerical rating scale (NRS) pain scores using an α of 0.05, a power of 0.80 and a medium effect size (*f* = 0.30). A sample size of 20 was required to detect a clinically important difference of a reduction in pain of two points on an NRS [21]. Participants were eligible if they met the ACR criteria for HOA [22], had normal or corrected-to-normal vision and had not received any new hand treatment in the 2 months preceding the experiment. All participants were naïve as to the purpose of the experiment and provided written consent. This study was conducted in accordance with the Declaration of Helsinki and ethical approval was obtained from the University of Nottingham (Ref: 578).

### Study design

The study used a two-period randomized crossover design consisting of two visits (stretch illusion visit and hand-only visit) with a 1-week washout period between. Visits were counterbalanced between participants and conditions were randomized within each visit.

### Apparatus

The current study used a MIRAGE mediated-reality device [23], presenting real-time physically coincident video using a setup of cameras and mirrors.

### Outcome measures

#### Subjective ratings of pain

The primary outcome measure was pain intensity on an 11-point NRS measured at baseline and after every condition. NRSs have good psychometric properties and are an appropriate tool in the therapeutic evaluation of OA [24, 25].

#### Subjective experience of the illusion

Subjective experience of the illusion was measured using a 7-point Likert scale (−3 = totally disagree; +3 = totally agree) on six statements relating to the emotional experience, perceived hand size, susceptibility, ownership, agency of the experimental hand and a control statement (see Table 1).

**Table 1:** Statements used to measure the subjective experience during the conditions

Category	Statements
Susceptibility	It felt as though my hand was being stretched.
Ownership	I feel like the hand I am looking at is my hand.
Agency	It feels as though my hand is in my control.
Perceived hand size	My hand feels longer than normal.
Affective experience	I do not like the way my hand looks now.
Control	I feel as though I have two right hands.

#### Quantitative sensory testing

Pain sensitivity was measured using PPTs, which investigate the relationship between sensory input and pain perception and provide an assessment of the state of the peripheral nervous system [26, 27]. PPTs were taken at baseline and immediately after each condition using a hand-held pressure algometer (Series 3; Mark-10, Compiague, NY, USA) with a 1 cm^2^ rubber tip at a rate of 0.1 kg/s. Consistent with previous studies [1], measurements were taken from the most painful part of the hand on the day of testing on the most affected hand (or dominant hand if both equally affected), the same point on the other hand and the sternum. After each condition, two measurements were taken from each measurement point, 15 s apart and in an alternating manner, with the mean of the two measurements being used for analysis [28]. Participants verbally indicated the moment the pressure became painful or unpleasant, upon which the experimenter immediately ceased pressure.

#### Measurement of hand function

The Functional Index for Hand OA (FIHOA) is a self-report measurement of hand function consisting of 10 items rated on a 4-point scale [29], with high internal consistency (α = 0.85–0.90) and test–retest reliability (intraclass correlation 0.74–0.95) [30, 31]. Statements include: ‘Are you able to turn a key in a lock?’ and ‘Are you able to clench your fists?’ Scores range from 0 to 30; with higher scores denoting worse hand function. The measurement was administered before and 48 h after each visit using a follow-up questionnaire.

### Procedure

**Fig. 1:**
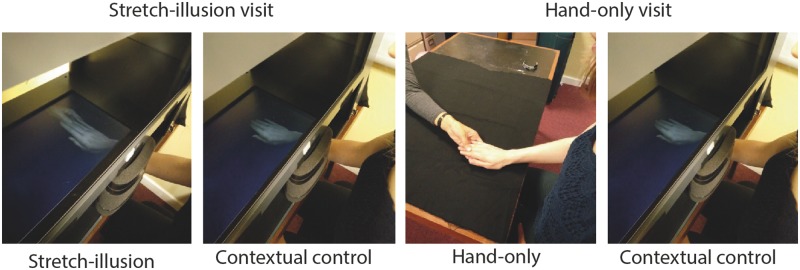
Experimental setup The two visits were counterbalanced between participants and the conditions were randomized in each visit. The contextual control was the same for both visits. Baseline measures were included in both visits (always first).

Participants attended twice, each visit comprising two conditions (Fig. 1), all conducted by the same experimenter. At baseline, participants completed several self-report measures of hand pain and hand function, were asked about their most affected hand, rated any pain using an NRS and underwent baseline PPT testing.

The stretch illusion condition involved participants viewing a live virtual representation of their own hand, in the same spatial location as their actual hand, being stretched visually at the same time as feeling their hand being pulled/stretched out. The visual stretch centred on the most painful part of the hand, identified prior to the start of the experiment. The stretch illusion is thought to be convincing, as it involves congruent visual, tactile and proprioceptive manipulation of the participant’s hand, giving the felt illusion of the hand being enlarged [32].

In the hand-only condition, the participant placed their most painful hand on a table in front of them. Identical to the stretch illusion condition, the experimenter took hold of the most painful part of the hand and pulled gently.

Both contextual control conditions involved the participant viewing their most painful hand inside MIRAGE without tactile or visual manipulation. The hand position, experimenter interaction, outcome measures and timings were kept identical to the stretch illusion condition and therefore were considered as contextual controls for the purposes of this study.

### Statistical analysis

SPSS 22.0 (IBM, Armonk, NY, USA) was used for statistical analyses. Data normality was assessed using Kolmogorov–Smirnov tests and outliers were identified by examination of studentized residuals for values >±3. Greenhouse–Geisser corrected values are reported if the assumption of sphericity was violated, as indicated by Mauchly’s test. PPT data were transformed to meet the assumptions of linear regression using the base 10 logarithmic transformation.

Subjective pain ratings were analysed using repeated measures ANOVA with condition (baseline, experimental, contextual control) and visit (illusion visit, hand-only visit) as within-subject factors, with the experimental condition referring to the stretch illusion and hand-only condition in the stretch illusion visit and hand-only visit, respectively. Significant interactions were followed up with separate repeated measures ANOVAs per visit. A paired samples *t*-test between baseline pain ratings tested whether baseline pain differed between visits. The potential for visit order to contribute to the variability in outcome responses on pain scores was investigated using a three-way mixed ANOVA with condition (experimental, contextual control) and time (pre-, post-) as the within-subject factors and visit order (start with illusion visit, start with hand-only visit) as the between-subject factor.

PPTs were analysed using a three-way mixed ANOVA, with the factor body site (most affected hand, other hand, sternum), condition (baseline, experimental, contextual control) and visit (stretch illusion visit, hand-only visit) as within-subject factors. Significant two-way interactions were investigated using one-way repeated measures ANOVAs for each visit separately.

Differences between the subjective experience of the illusion were investigated using multivariate ANOVA for repeated measures with condition (stretch illusion, hand only and both contextual controls) as within-subject factors on the dependent variables (affective experience, perceived hand size, susceptibility, ownership, agency, control). Significant interactions were followed up with univariate ANOVAs. FIHOA scores were analysed using a repeated measures ANOVA with visit (stretch illusion visit, hand-only visit) and time (pre-visit, 48 h) as within-subject factors.

Pearson correlations examined relationships between current pain at the start of the visit and illusion strength on ownership and susceptibility subscales to investigate individual variability and susceptibility to the illusion [5, 33].

## Results

### Demographic data

All participants had hand pain due to primary OA of the DIP or PIP joints, with involvement of other joints including the hip, knee and ankle. One participant was excluded due to technical issues and one participant dropped out due to a significant change in medication. Thus 28 participants were included for analysis (Table 2).

**Table 2:** Participant characteristics (*n* = 28)

Participants, *n*	28
Age, years	70.50 (7.85)
Female, *n*	21
Right hand dominant, *n*	22 (2 ambidextrous)
OA in other joints (pain location), *n*	
Left hand	2
Right hand	1
Bilateral	25
No pain	0
Most affected hand, *n*	
Right hand	12
Left hand	6
No difference	10
Occurrence of hand pain in the past 12 months, *n*	
<3 months	3
≥3 months	25
First onset of hand pain, *n*	
Within the last 12 months	1
1–<5 years	4
5–<10 years	11
≥10 years	13
Average pain intensity both visits	4.55 (2.03)
Pain intensity, stretch illusion visit^a^	
Current pain (0–10)	4.39 (2.66)
Pain over the last 48 h	5.86 (2.19)
Overall hand pain at follow-up	5.44 (2.04)
Pain in all joints in the last 48 h	5.93 (2.73)
Function stretch illusion visit	
Satisfaction with function at 48 h^b^	5.68 (2.20)
FIHOA (0–30)^c^	11.68 (4.80)
Pain intensity, hand-only visit^a^	
Current pain (0–10)	4.71 (2.43)
Pain over the last 48 h	5.86 (2.09)
Pain in all joints in the last 48 h	5.92 (2.0)
Function hand-only visit	
Satisfaction with function in the last 48 h^b^	5.14 (2.21)
FIHOA (0–30)^c^	11.75 (5.52)

All values are mean (s.d.) unless otherwise specified.

^a^Pain intensity measured on a 0–10 NRS.

^b^0 = very satisfied, 10 = not at all satisfied.

^c^Higher scores denote decreased hand function. Current pain refers to pain at the start of the stretch illusion visit or the hand-only visit.

### Subjective pain ratings

**Fig. 2:**
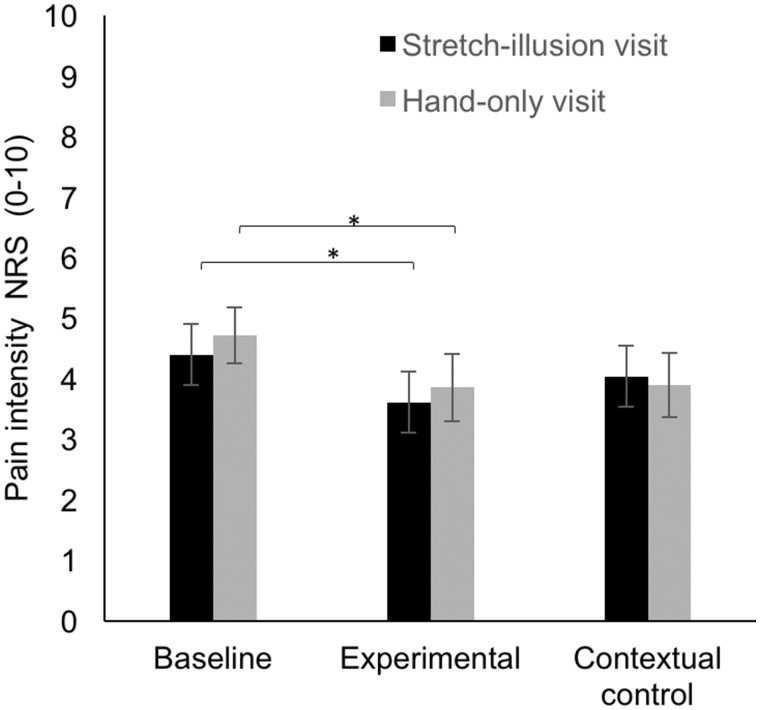
The mean effect of condition on pain intensity (NRS ratings) NRS scores were significantly lower after the experimental condition compared with the baseline for both visits. The experimental condition refers to the stretch illusion and hand-only condition in the stretch illusion visit and hand-only visit, respectively. Error bars represent standard error. *Statistically significant difference between conditions after Bonferroni corrections.

Mean pain ratings are displayed in Fig. 2. There was a main effect of condition [*F*(1.510, 54) = 4.364, *P* = 0.028, η^2^ = 0.139], but no main effect of visit order [*F*(1, 27) = 0.130, *P* = 0.722, η^2^ = 0.005] and no significant two-way interaction between condition and visit [*F*(1.596, 43.081) = 0.319, *P* = 0.728, η^2^ = 0.012]. NRS pain scores were significantly lower in the experimental condition compared with baseline [*F*(2, 26) = 5.500, *P* = 0.013, η^2^ = 0.297], with a mean difference of 0.82 (95% CI 0.151, 1.492). NRS scores were not significantly different between contextual controls [*M* = 3.964 (95% CI 3.013, 4.915)] and baseline [*M* = 4.554 (95% CI 3.751, 5.356)] [*F*(2, 26) = 5.500, *P* = 0.332, η^2^ = 0.302] or between the experimental condition [*M* = 3.732 (95% CI 2.807, 4.657)] and contextual control. A sensitivity analysis including only people with baseline scores >3 on both visits yielded similar results. Follow-up questionnaire data were collected from 26 of the 28 participants after the illusion visit and 25 participants after the hand-only visit. For the majority of participants, any pain reduction lasted only for the duration of the experiment. Some participants reported a decrease in pain lasting up to 60 min after the visit and a small percentage of participants had an increase in pain after the visit (supplementary File S1, available at *Rheumatology Advances in Practice* online).

### PPTs

**Fig. 3:**
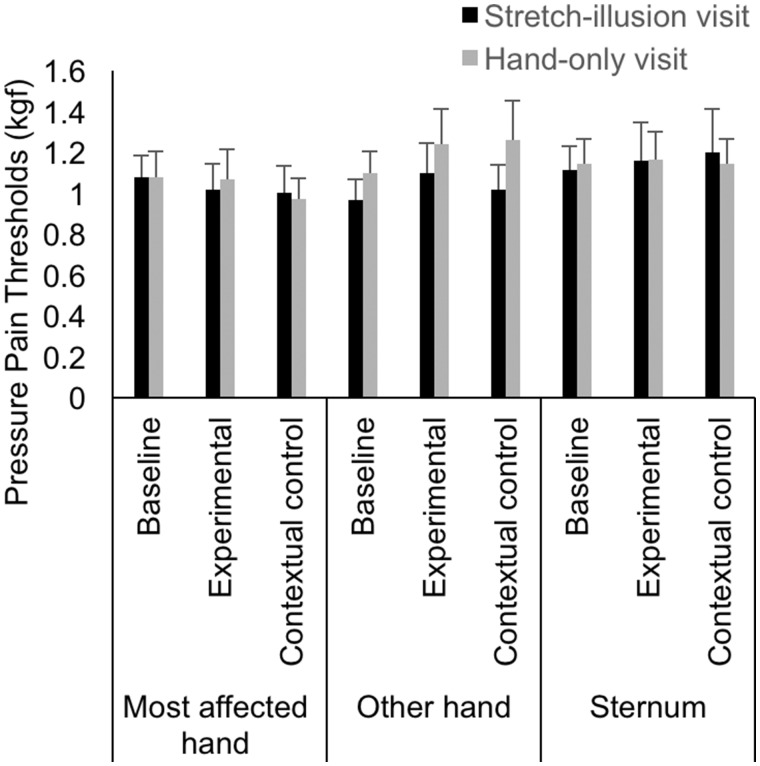
Mean pressure pain thresholds (kgF) after each condition An anti-log of the mean of the log_10_ transformed values was conducted to present the results as a mean. The experimental condition refers to the stretch illusion and hand-only condition in the stretch illusion visit and hand-only visit, respectively. Error bars represent the standard error of the mean.

Mean PPTs in each condition are displayed in Fig. 3. PPT measurements were taken from the most painful point on the affected hand and included the DIP (18% participants), PIP (29%), thumb IP (4%) and thumb CMC (39%) joints. There was a main effect of body site [*F*(2, 50) = 3.416, *P* = 0.041, η^2^ = 120], a significant interaction between body site and condition [*F*(4, 100) = 2.73, *P* = 0.033, partial η^2^ = 0.099] and a marginal interaction between body site, condition and visit [*F*(4, 100) = 2.357, *P* = 0.059, η^2^ = 0.086]. There was no significant effect of condition [*F*(1.378, 34.451) = 1.720, *P* = 0.199, η^2^ = 0.064] or visit [*F*(1, 25) = 0.233, *P* = 0.634, η^2^ = 009] and no significant interaction between body site and visit [*F*(2, 50) = 1.245, *P* = 0.297, η^2^ = 047] or condition and visit [*F*(2, 50) = 0.948, *P* = 0.395, η^2^ = 0.037].

### Subjective experience of the illusion

**Fig. 4:**
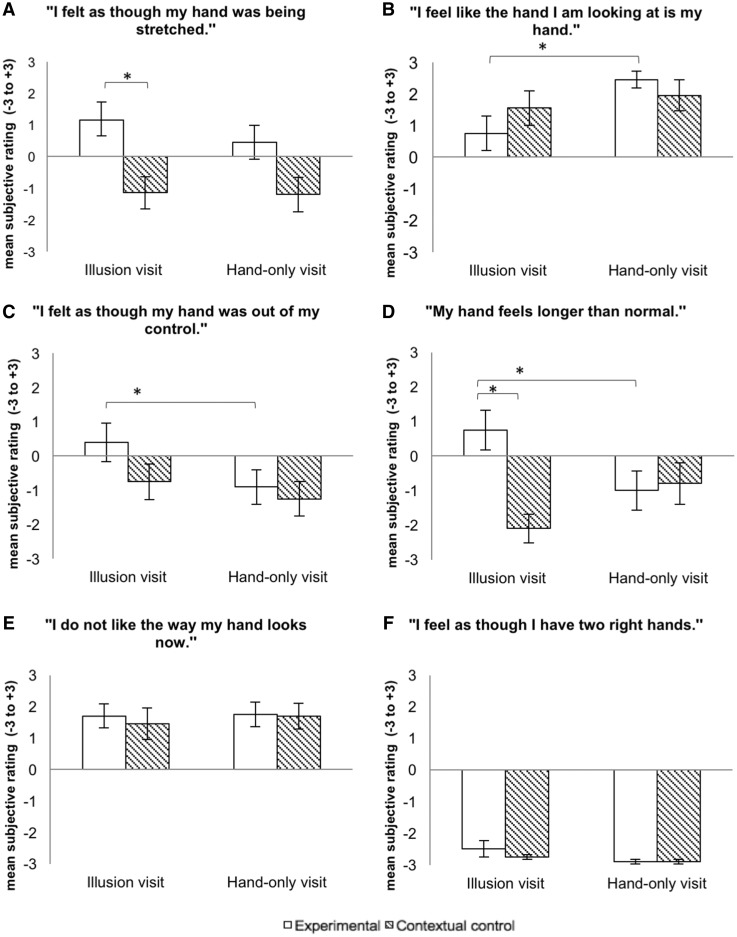
Mean objective ratings of the statements for the illusion visit and the hand-only visit between experimental conditions and contextual controls The experimental condition refers to the stretch illusion in the stretch illusion visit and hand-only condition in the hand-only visit. The contextual control was the same for both visits and involved the participant looking at their most painful hand inside MIRAGE without any tactile or visual manipulation. Error bars are standard error. *Statistically significant difference between conditions after Bonferroni correction. −3 = no, not at all; +3 = yes, lots and lots.

The multivariate ANOVA showed a statistically significant difference between conditions on the combined dependent variables [*F*(18, 147.563) = 3.325, *P* < 0.001, Wilks’ Λ = 0.390, η^2^ = 0.269], suggesting that illusion strength was not the same for the stretch illusion, hand-only and contextual controls (Fig. 4). Follow-up univariate ANOVAs showed that there were statistically significant differences in perceived hand size (*P* < 0.001), susceptibility (*P* < 0.001), ownership (*P* = 0.010) and agency (*P* = 0.017), but not affective experience (*P* = 0.886) or in responses to the control question (*P* = 0.308). *Posthoc* analysis using unpublished data from 17 participants without OA [mean age 63.6 years (s.d. 3.75), six female] was conducted to determine if the high levels of dissatisfaction differed between groups. People with HOA presented a greater dislike of the hand during contextual control [*M *=* *1.79 (s.d. 1.64)] compared with the healthy control group [*M* = 0.47 (s.d. 1.94)], a statistically significant difference [*M* = 1.31 (95% CI −2.405, −2.248), *t*(43) = −2.433, *P* = 0.019, *d* = 0.77]. There was no difference in dislike after stretch illusion between the HOA group [*M* = 1.64 (s.d. 1.93)] and the healthy control group [*M* = 2.00 (s.d. 1.27)], *M* = 36 (95% CI −0.71, 1.42), *t*(43) = 0.677, *P* = 0.50, *d* = 0.22].

Illusion susceptibility was highest after the stretch illusion, which was significantly different from contextual control (*P* < 0.001) but not the hand-only condition (*P* = 0.253) (Fig. 4A). Ownership of the hand was lowest after the stretch illusion and significantly lower compared with the hand-only condition (*P* < 0.001), with no significant difference compared with contextual control (*P* = 0.112) (Fig. 4B). There was a significant loss of agency after the stretch illusion compared with the own-hand condition (*P* = 0.030) (Fig. 4C). However, this was not significantly different from contextual control (*P* = 0.059). Participants reported their hand/finger as feeling longer after illusory stretching of the finger compared with contextual control (*P* < 0.001) and hand only (*P* = 0.012) (Fig. 4D).

### FIHOA

There was no effect of visit [*F*(1, 24) = 1.20, *P* = 0.733] or time [*F*(1, 24) = 0.210, *P* = 0.651] on FIHOA scores between baseline and the 48-h follow-up and no interaction between visit and time [*F*(1, 24) = 1.231, *P* = 0.278].

### Correlations between baseline pain and other outcome measures

Current pain at the start of the stretch illusion visit (P1) and current pain at the start of the hand-only visit (P2) were strongly correlated (all *P* < 0.01; see supplementary File S2, available at *Rheumatology Advances in Practice* online), suggesting high test–retest stability and reliability of the individual pain measures and between visits within subjects. P1 and P2 strongly correlated with all other pain and function ratings made on the day (all *P* < 0.05) except for hand pain/aching or stiffness over the last month (*P *> 0.05). P2 did not correlate significantly with hand function or FIHOA scores (all *P *> 0.05). There was no significant correlation between P1 or P2 and change in pain after the stretch illusion (*P* > 0.05). P1 was negatively correlated with ownership of the hand after the stretch illusion [Pearson’s *r*(30) = −0.41, *P* = 0.01]. That is, the greater the pain ratings at baseline, the lower the ownership of the hand after the stretch illusion. *Posthoc* analysis showed a similar but non-significant correlation between P1 and ownership after the contextual control [*r*(29) = −0.28, *P* = 0.07].

There was no significant correlation between P2 and ownership after the hand-only condition in the hand-only visit [Pearson’s *r*(27) = −0.10, *P* = 0.29].

There was a small positive correlation between P1 and susceptibility to the stretch illusion [Pearson’s *r*(28) = −0.30, *P* = 0.05] in that the greater the pain ratings at baseline, the higher the susceptibility to the stretch illusion. There was a moderately positive relationship between current pain and the feeling that the hand/finger was stretched after the hand-only condition [Pearson’s *r*(24) = −0.43, *P* = 0.027]. Finally, there was a negative correlation between ownership and susceptibility, in that higher ownership was related to lower susceptibility to the illusion [Pearson’s *r*(28) = −0.32, *P* = 0.038].

## Discussion

This study investigated the role of contextual factors underlying modulation of pain and hand function during multisensory illusions in people with painful HOA. The data show three main findings: First, stretching while viewing both the real and virtual hand led to a reduction in pain compared with baseline. Second, only virtual stretching of the hand led to changes in perceived hand size. Finally, existing pain predicts susceptibility to the illusion and reduction of ownership (see Nierula *et al.* [34] for similar observations) but does not predict analgesic efficacy.

The finding of an analgesic effect after both stretch conditions is interesting because while recent evidence suggests that vision of both the virtual and real body is found to reduce pain [15–17, 34] and that this is specific to viewing one’s own body [8], the current study found that this is not necessarily the case when contextual cues are accounted for. Viewing the body can modulate the sensory processing of pain but has been shown to depend on the specific visual context in which it occurs, depending on whether the painful body is presented as smaller or larger [35, 36]. Although there was a significant reduction in pain after the experimental conditions compared with baseline, they did not significantly differ from contextual control, suggesting that although contextual factors play an important part, the reduction in pain might be strengthened by the feeling of ownership and perceived body size or even sensory feedback. Thus the findings of the current study go beyond previous findings as they demonstrate that multisensory illusions applied to the painful hand produce analgesia beyond that of merely viewing the limb.

A loss of body ownership over the painful limb has been suggested as a possible mechanism for the analgesic effects of the illusion [37] and is supported by findings in experimental studies in which visually changing the size or shape of the affected limb can reduce the pain and swelling associated with a reduction of ownership over that limb [7], as well as physical symptoms [38, 39]. The finding of no analgesic effect in the contextual control condition in which ownership remained high lends further support for this theory. However, ownership over a virtual limb does not necessarily lead to a loss of ownership over the actual limb [40, 41]. Furthermore, our findings show that although pain predicts a reduction of ownership over the painful hand, it is not necessary for analgesic effects to occur when viewing the hand directly. Future studies are needed to investigate what happens to the ‘real’ affected painful hand in terms of body ownership and whether this affects pain.

The findings demonstrate a positive correlation between the amount of pain at baseline and susceptibility to the illusion, in that the greater the pain ratings, the greater the perceived feeling of stretch after the stretch illusion. Variability in susceptibility to bodily illusions has been reported previously [5, 33] and is positively correlated to analgesic efficacy [42]. However, our findings show that although pain may predict susceptibility, susceptibility does not lead to greater analgesia in the stretch illusion condition. This suggests that one does not need to be susceptible to an illusory change in body shape for analgesic effects to occur. These results highlight the need for more research into the causal relations between body image disturbances and pain [7, 10].

The different conditions had no effect on the affective experience of the hand. Instead, the findings demonstrate strong dislike of the hand across all conditions. Indeed, even when just looking at the hand, people with HOA present with a high level of body dissatisfaction compared with healthy controls. In contrast, while illusory stretching decreased body satisfaction in healthy participants, no change was observed in the HOA group, indicating that HOA participants may present with abnormally high body dissatisfaction under normal conditions. There is a growing body of evidence suggesting that body image—a person’s perception of their body—is disrupted in chronic pain, including OA [4]. Another important factor impacting on body image is the deformities often associated with HOA. The dissatisfaction experienced by individuals living with this condition has been found to have a major functional and psychological impact on everyday life [11]. Future work should therefore address the significant body dissatisfaction in these conditions and how this might contribute to body image and the maintenance of persistent pain.

Several limitations of the methods and results deserve consideration. First, to minimize the burden on participants, the number of conditions and repetition of PPT testing was minimized. Although the current design allowed for a comparison between the view of the hand in or outside the virtual environment and with or without any tactile and visual manipulation, it is difficult to disentangle the tactile contribution from the observed analgesic effects. Second, it is possible that stretching the hand is in itself analgesic. The current research is intended as a starting point for further research in what may be a promising means of alleviating pain in HOA and requires further investigating in larger samples and in other chronic pain conditions.

The current study highlights the importance of the context in which pain occurs; in this case, virtual and physical stretching modulating pain but not viewing the hand alone in the virtual environment. Although the experimental conditions were not different from contextual control in terms of analgesic effects, the results provide evidence for a change in perceived hand size, ownership and agency after stretching the virtual body. Furthermore, while pain at baseline predicted illusion strength, it did not predict analgesic efficacy, which could possibly explain the large variability in response to the illusion observed in this study and in other body ownership paradigms.

## Supplementary Material

Supplementary Data
