# Supplementary material for: An investigation of contextual factors in the application of multisensory illusions for analgesia in hand osteoarthritis
Source: Rheumatol Adv Pract. 2018 Jul 21;2(2):rky019. doi: 10.1093/rap/rky019 (PMC6649980; doi:10.1093/rap/rky019)
Supplement: Supplementary Data [file rky019_supp.zip › Themelis Supplementary File 1.docx]

**Supplementary File 1**

Change in subjective pain ratings after both visits

|  | No change in pain at all | Decrease <15 min. after visit | Decrease <30 min. after visit | Decrease <60 min. after visit | Decrease <90 min. after visit | Decrease <24h after visit | Increase after visit |
| --- | --- | --- | --- | --- | --- | --- | --- |
| Follow up Stretch-illusion visit | 23/26 (88%) | 1/26  (4%) | - | 1/26  (4%) | - | - | 1/26  (4%) |
| Follow up hand-only visit | 21/25  (84%) | 2/25  (8%) | 1/25  (4%) | - | - | - | 1/25  (4%) |
