# Supplementary material for: An investigation of contextual factors in the application of multisensory illusions for analgesia in hand osteoarthritis
Source: Rheumatol Adv Pract. 2018 Jul 21;2(2):rky019. doi: 10.1093/rap/rky019 (PMC6649980; doi:10.1093/rap/rky019)
Supplement: Supplementary Data [file rky019_supp.zip › Themelis Supplementary File 2.docx]

| **Measure** | **Correlation (*r)***  ***Visit 1&2*** | ***p*** |
| --- | --- | --- |
| **Single pain intensity ratings** |  |  |
| Current pain most painful joint | **.31*** | **.049** |
| Overall hand pain last 2 weeks | **.752**** | **<.001** |
| Overall hand pain last 48h | **.709**** | **<.001** |
| Most painful joint 48 hours | **.666**** | **<.001** |
| Most painful joint last 2 weeks | **.607**** | **<.001** |
| Activity arthritis 48 hours | **.765**** | **<.001** |
| Thumb pain 48 hours | **.752**** | **<.001** |
| Pain in all joints 48 hours | **.693**** | **<.001** |
| **Hand function** |  |  |
| Satisfaction with function 48 hours | **.372*** | **.025** |
| Hand pain/aching or stiffness over the  last month | **.573**** | **<.001** |
| FIHOA | **.737**** | **<.001** |

**Supplementary File 2**

Pearson Correlations between subjective pain ratings baseline 1^st^ visit and baseline 2^nd^ visit n=28. (significant correlation is highlighted in bold).

** Correlation is significant at the .01 level

* Correlation is significant at the .05 level
